# Time dependent differential regulation of a novel long non-coding natural antisense RNA during long-term memory formation

**DOI:** 10.1038/s41598-021-83190-4

**Published:** 2021-02-11

**Authors:** Sergei Korneev, Jekaterina Garaliene, Gabriella Taylor, Ildikó Kemenes, György Kemenes

**Affiliations:** Sussex Neuroscience, School of Life Sciences, University of Sussex, Brighton, BN1 9QG UK

**Keywords:** Learning and memory, Classical conditioning, Molecular biology, Non-coding RNAs, Long non-coding RNAs, Neuroscience, Molecular neuroscience

## Abstract

Long natural antisense transcripts (NATs) have been demonstrated in significant numbers in a variety of eukaryotic organisms. They are particularly prevalent in the nervous system suggesting their importance in neural functions. However, the precise physiological roles of the overwhelming majority of long NATs remain unclear. Here we report on the characterization of a novel molluscan nitric oxide synthase (NOS)-related long non-coding NAT (*Lym-NOS1AS*). This NAT is spliced and polyadenylated and is transcribed from the non-template strand of the *Lym-NOS1* gene. We demonstrate that the *Lym-NOS1AS* is co-expressed with the sense *Lym-NOS1* mRNA in a key neuron of memory network. Also, we report that the *Lym-NOS1AS* is temporally and spatially regulated by one-trial conditioning leading to long term memory (LTM) formation. Specifically, in the cerebral, but not in the buccal ganglia, the temporal pattern of changes in *Lym-NOS1AS* expression after training correlates with the alteration of memory lapse and non-lapse periods. Our data suggest that the *Lym-NOS1AS* plays a role in the consolidation of nitric oxide-dependent LTM.

## Introduction

The gaseous signalling molecule nitric oxide (NO) has been implicated in the regulation of a number of important neurophysiological processes such as neurogenesis, sleep–wake cycle, appetite, hormone release, and blood pressure^1^. Of particular interest is the discovery that NO plays a significant role in the early stages of memory formation in a variety of species from humans to invertebrates^2–5^. In the snail, *Lymnaea stagnalis*, a well-established model organism for learning and memory studies, there is an obligatory requirement for NO during the first 5 h of long-term memory (LTM) formation following single-trial associative food-reward conditioning^6^. On the other hand, NO is a highly reactive free radical with potential cytotoxic properties. Indeed, inappropriate changes in the level of NO contribute to the development of serious pathological conditions of the nervous system^7,8^. Therefore, NO production in the normal brain is tightly regulated through a variety of mechanisms. Long non-coding Natural Antisense Transcripts (NATs) appear to be one of the most intriguing additions to the list of such mechanisms.

Long NATs collectively refer to endogenous RNA molecules with lengths exceeding 200 nucleotides that are complementary to RNA transcripts of already established function. Depending on their origin, all long NATs can be grouped into two classes: *cis*-encoded and *trans*-encoded. *Cis*-encoded NATs are transcribed from the same loci as their sense counterparts whereas *trans*-encoded NATs are transcribed from different loci. Recent studies have shown that long NATs are abundant in eukaryotes and are particularly prevalent in the central nervous system^9–12^.

In our previous publications we reported on the discovery of two *trans*-encoded long NATs (*antiNOS-1* and *antiNOS-2*), which are expressed in the brain of the pond-snail, *Lymnaea stagnalis,* and are complementary to the nitric oxide synthase (NOS)-encoding mRNA^13–16^. Both NATs are transcribed from a NOS pseudogene and are associated with the negative regulation of the production of gaseous neurotransmitter nitric oxide (NO) by NOS.

In this study we report on the characterization of a novel *cis*-encoded NAT, which is transcribed from the non-template strand of the *Lym-NOS1* gene. Hereafter we will refer to this antisense RNA as *Lym-NOS1AS*. The *Lym-NOS1AS* is spliced, polyadenylated, does not contain ORFs larger than an arbitrary size and could therefore be assigned to a group of long non-coding RNAs (ncRNAs). We demonstrate that the *Lym-NOS1AS* is co-expressed with the *Lym-NOS1* mRNA in the cerebral giant cell (CGC), a neuron with an established role in the conditioned feeding response^17^. Furthermore, we report on the timed and targeted differential regulation of *Lym-NOS1AS* in the brain by reward conditioning leading to LTM formation. Intriguingly, these learning-induced changes in the expression of *Lym-NOS1AS* correlate well with the previously discovered alteration of memory lapse and non-lapse periods^18^.

## Results

### A *cis*-encoded long NAT complementary to *Lym-NOS1* mRNA is expressed in the brain

**Figure 1:**
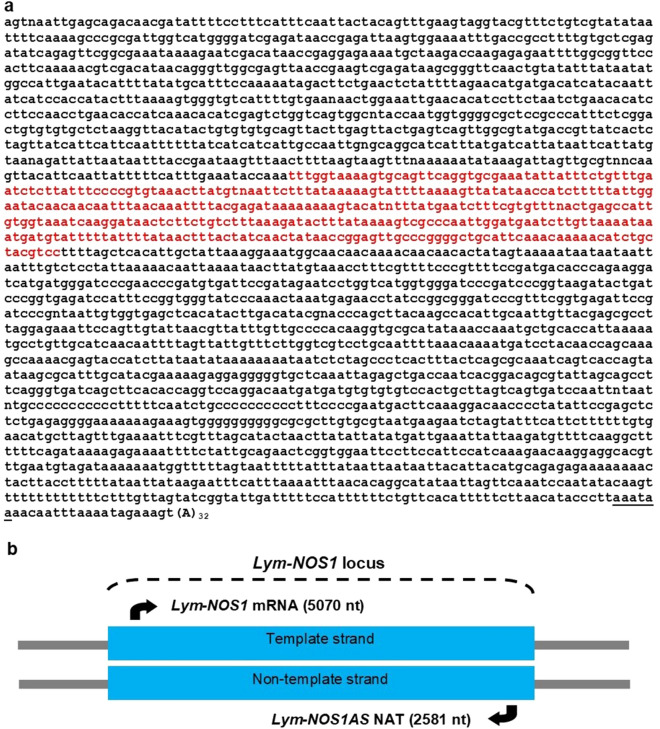
*Lym-NOS1AS* is a long *cis*-encoded natural antisense transcript. (**a**) Nucleotide sequence of *Lym-NOS1AS* NAT (accession number MW300420). The antisense region (shown in red) is complementary to *Lym-NOS1* mRNA (accession number AF012531). A putative polyadenylation signal is underlined. (**b**) A schematic diagram showing that *Lym-NOS1AS* is transcribed from the non-template strand of the *Lym-NOS1* locus.

While screening a snail CNS cDNA library with a NOS-specific probe, we isolated a transcript of about 2500 nt in length. Although the transcript possesses some features of a typical mRNA, such as the polyadenylation signal and a poly(A) tail, it is unlikely that it can be translated because of the presence of multiple stop codons in all the reading frames. This indicated that we had cloned a long ncRNA. Another and rather unexpected feature of this novel ncRNA was the presence of 367 nt sequence, which is complementary to the 5′ end of the *Lym-NOS1* mRNA (Fig. 1a). The degree of complementarity (100%) indicated strongly that this ncRNA is transcribed from the non-template strand of the *Lym-NOS1* locus and belongs to a group of long *cis*-encoded NATs (Fig. 1b). Consequently, we named this NAT *Lym-NOS1AS* (accession number MW300420).

**Figure 2:**
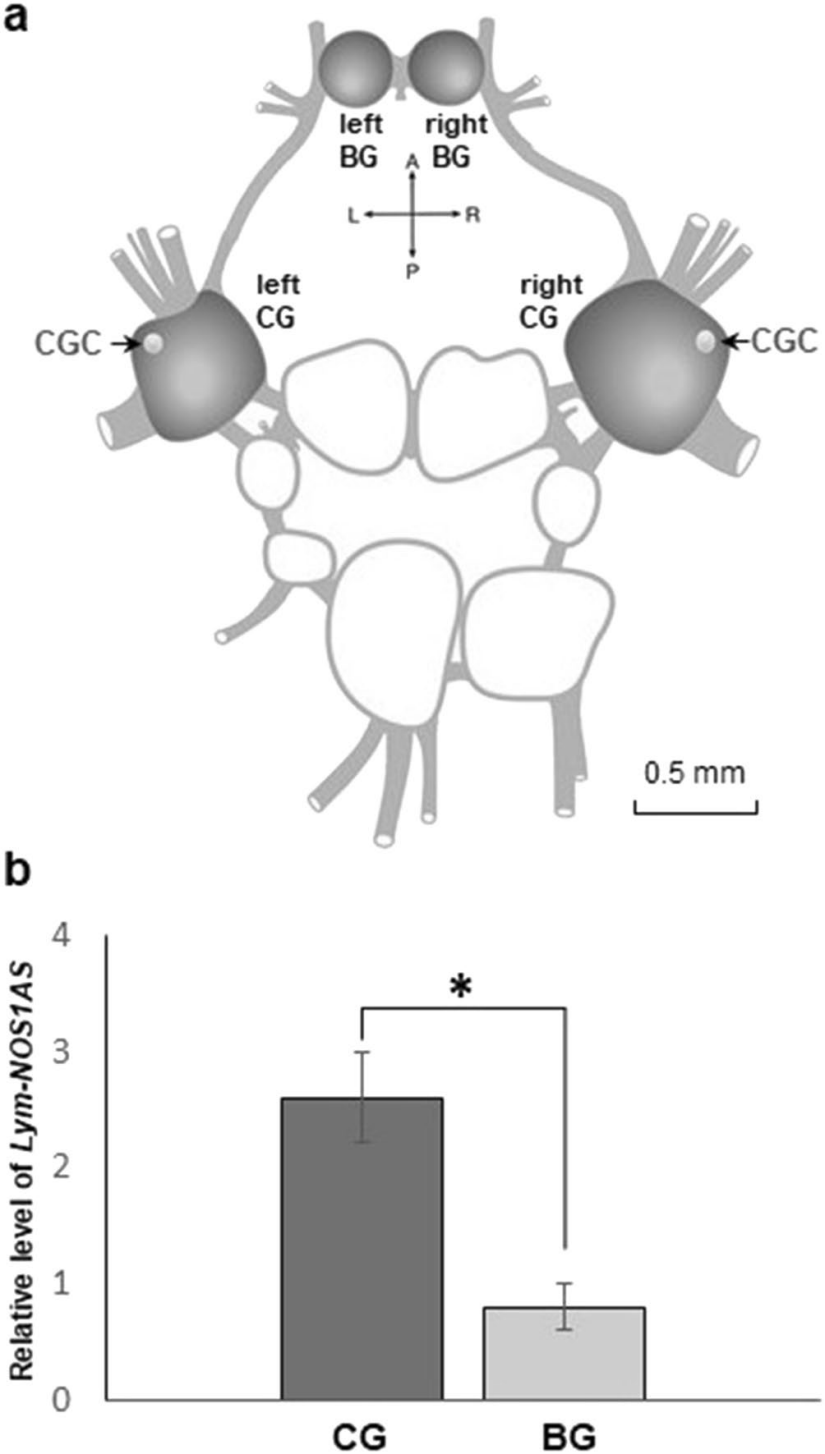
*Lym-NOS1AS* expression in ‘naïve’ ‘learning’ ganglia. (**a**) A diagram of the *Lymnaea* CNS. The ‘learning’ ganglia [cerebral ganglia (CG) + buccal ganglia (BG)] are highlighted in grey. The white dots in the cerebral ganglia indicate the position of the paired Cerebral Giant Cells (see Fig. 3). (**b**) Results of real-time RT-PCR performed on the cerebral (dark grey) and buccal (light grey) ganglia dissected from naïve snails (n = 5 samples, each sample contained material from 4 animals). The relative level of *Lym-NOS1AS* expression was calculated as 2^−ΔΔCt^. The asterisk indicates a significant difference between the two groups (two-tailed t-test for independent samples*: t* = −4.58, *df* = 8, *p* < 0.01). The data in this figure are shown as means ± SEM.

Of note, the central nervous system of *Lymnaea* contains 11 ganglia (paired cerebral, pedal, pleural, parietal, buccal ganglia and unpaired visceral ganglion, Fig. 2a). Our previous work established that the implicit memory trace resulting from single-trial food-reward classical conditioning is both acquired and stored in the same neural circuit located in the buccal and cerebral ganglia (hereafter ‘learning ganglia’)^19^. With this in mind, we decided to determine whether the *Lym-NOS1AS* is differentially expressed in the two parts of the learning ganglia. To achieve this, we extracted RNA from individual cerebral or buccal ganglia and the purified RNA samples were then subjected to real-time RT-PCR to estimate the level of *Lym-NOS1AS* expression. The results of the analysis show that the expression level of the NAT is almost 3 times higher in the cerebral ganglia than in the buccal ganglia (Fig. 2b).

### *Lym-NOS1AS *NAT and *Lym-NOS1* mRNA are co-expressed in the CGC

**Figure 3:**
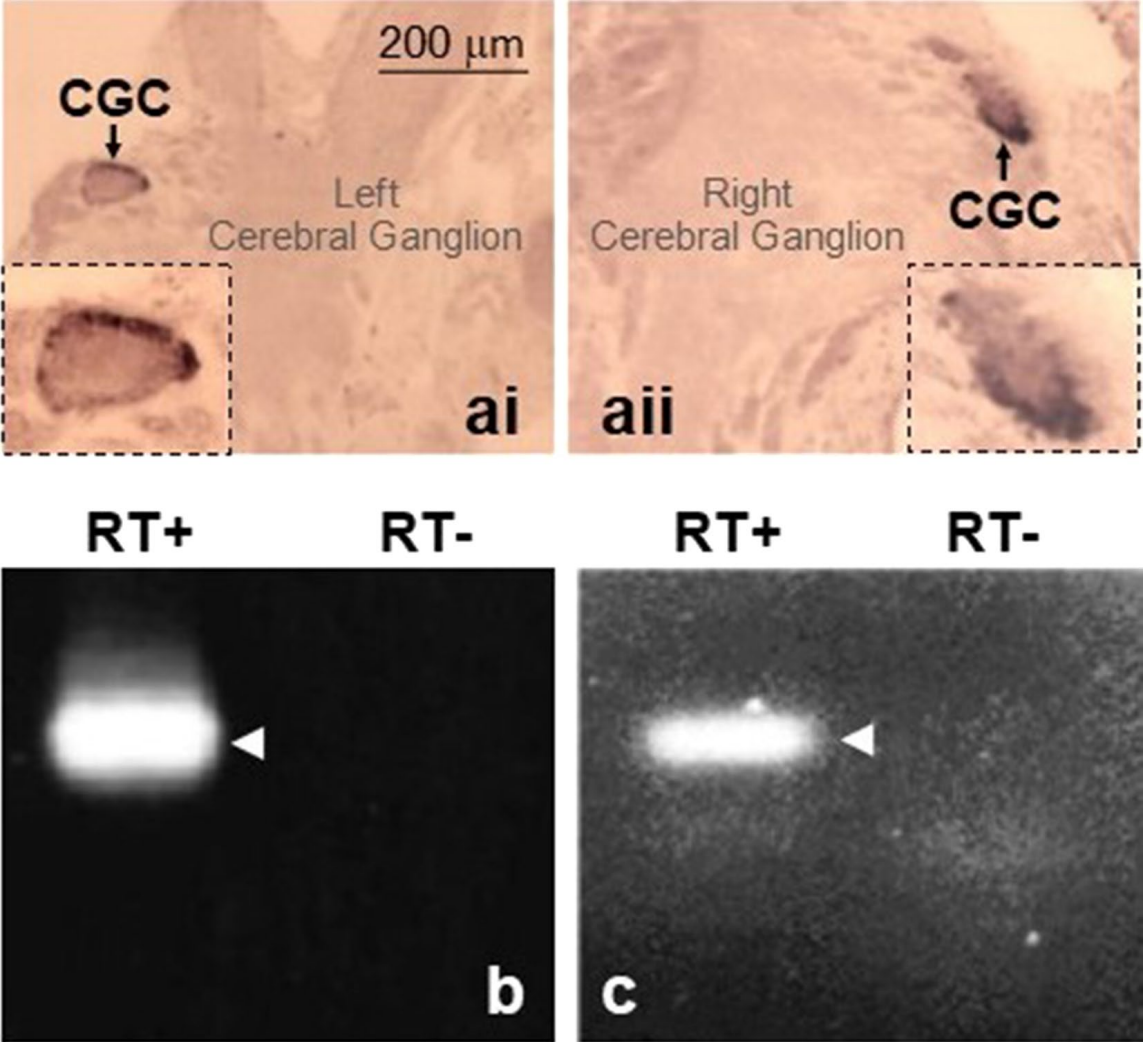
*Lym-NOS1* mRNA and *Lym-NOS1AS* NAT are expressed in the CGC. (**a**) In situ hybridization shows a bilaterally symmetrical pair of large neuronal cell bodies in the left (ai) and right (aii) cerebral ganglia (see Fig. 2a for location of the cerebral ganglia in the brain) stained with the *Lym-NOS1* mRNA specific probe. The cell bodies correspond in size and position to the identified CGCs (arrowed). Of note, there are several thousands of other types of neurons in the cerebral ganglia, but they are poorly stained, indicative of a very low level of the *Lym-NOS1* mRNA. The dotted line boxes show the stained CGCs at a higher magnification. It is worth mentioning that the CGC has a very large nucleus that fills a significant part of the cell but only the cytoplasm shows significant hybridization, as expected. (**b**, **c**) The results of conventional RT-PCR experiments conducted on RNA extracted from isolated CGCs to detect *Lym-NOS1* (**b**) and *Lym-NOS1AS* (**c**). The ‘RT+’ lanes show that the PCR products (arrowed) of the expected sizes (101 bp in case of *Lym-NOS1* and 108 bp in case of *Lym-NOS1AS*) are detected indicating that the CGCs express both *Lym-NOS1* and *Lym-NOS1AS*. The ‘RT−’ lanes represent the outcome of the control experiments in which reverse transcriptase was omitted. The absence of the amplification products in these lanes is indicative that the RNA preparation was free of DNA contamination.

In a previous work, we demonstrated that in rodents, a very similarly organized NOS-related *cis*-encoded NAT (*Mm-antiNos1*) acts as a negative post-transcriptional regulator of neuronal NOS gene expression^20^. This suggested to us that the *Lym-NOS1AS* can function in the same manner as the mouse ortholog regulating NO signaling in the brain. Apparently, however, it is only possible if both sense and antisense transcripts are co-expressed in a neuron. Hence, the question is: “Do such neurons exist in *Lymnaea* CNS?” Considering that *Lym-NOS1* mRNA is expressed only in a small population of neurons^21^, this issue deserves special attention. Therefore, we relied on an important advantage of our model system, which is the presence of large easily identifiable neurons. A particularly promising candidate that can be used to address the question is the pair of CGCs. First, our in situ hybridization experiments show that the CGCs display much higher level of *Lym-NOS1* mRNA in comparison to the other cell types located in the cerebral ganglia (Fig. 3ai, aii). Second, they are key neurons of the *Lymnaea* memory network^17^. Consequently, we identified and dissected 10 CGCs and RNA isolated from these cells was subjected to RT-PCR. The results of the analysis demonstrate clearly that the PCR products of exactly the expected sizes are detected when *Lym-NOS1* (Fig. 3b) or *Lym-NOS1AS* (Fig. 3c) specific primers were used. Of note, the identity of the PCR products was confirmed by cloning and sequencing. Thus, we can conclude that the CGCs are an example of neurons, which express both *Lym-NOS1* mRNA and *Lym-NOS1AS*.

### Single trial conditioning differentially regulates the expression of *Lym-NOS1AS* in the brain

Our discovery that *Lym-NOS1* mRNA and *Lym-NOS1AS* are co-expressed in the CGCs suggests that the *Lym-NOS1AS* is a part of the pathway involved in LTM formation. In order to test this hypothesis we conducted the following quantitative experiments.

**Figure 4:**
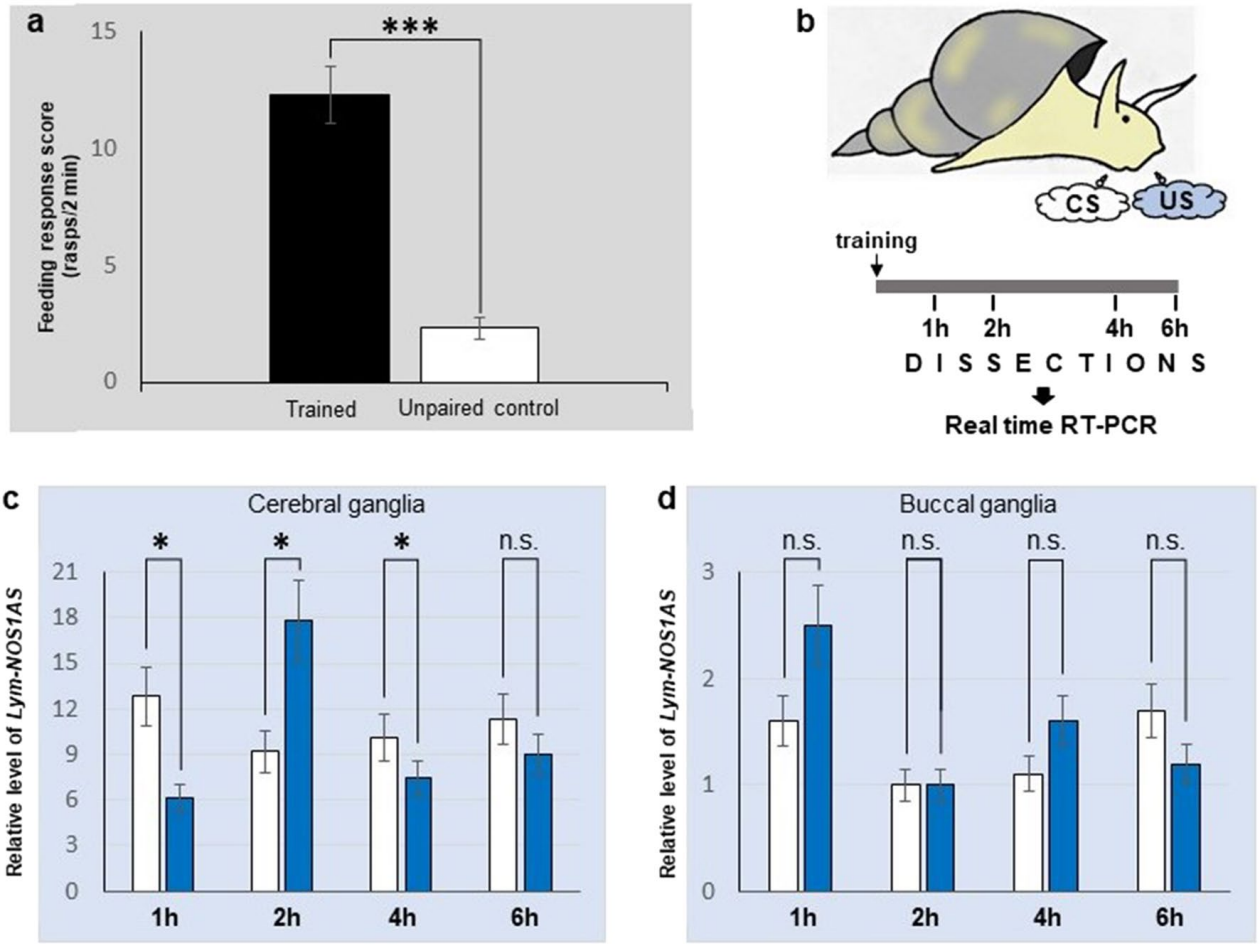
Training-induced differential regulation of the *Lym-NOS1AS*. (**a**) The result of the behavioural test of LTM formation at 24 h after training. The mean feeding response to amyl acetate (the CS) of the classically conditioned animals (black bar) is significantly higher than the response of the unpaired control animals (white bar) (n = 15 animals per group, two-tailed t-test for independent samples: *t* = 6.94, *df* = 28, *p* < 0.0001). (**b**) Schematic representation of the experiment to investigate whether single-trial reward conditioning is associated with timed and targeted changes in the expression of *Lym-NOS1AS*. (**c**, **d**) Results of real-time RT-PCR performed on the cerebral and buccal ganglia, respectively. The relative level of *Lym-NOS1AS* expression in ganglia from conditioned (blue bars) and unpaired control animals (white bars) dissected at 1 h, 2 h, 4 h and 6 h after training was calculated as 2^−ΔΔCt^. All data in this figure are shown as means ± SEM. Asterisks indicate significant differences (*p* < 0.05) between the conditioned and unpaired data at the same time point (n = 20 animals per group, unpaired two-tailed t-tests with Welch’s correction).

First and foremost, in all our experiments in which the effects of single trial conditioning on gene expression were studied, a randomly chosen group of animals was retained, trained and tested for LTM formation 24 h after training. This was to confirm that LTM would have occurred in the experimental animals that were used in our quantitative assays. Notably, the mean feeding response to amyl acetate (CS) of the trained snails was always significantly higher than the response of unconditioned animals (Fig. 4a).

To examine the effect of behavioral conditioning on temporal expression of the *Lym-NOS1AS* in different parts of the ‘learning ganglia’ we dissected individual cerebral and buccal ganglia at 1 h, 2 h, 4 h and 6 h after training (Fig. 4b). These time points were chosen because previous studies showed that (1) NO is only required for up to 5 h for successful consolidation of 24-h long-term memory after single-trial classical conditionin ^6^; (2) a period of memory lapse occurs at 2 h post-training, while at 1 h, 4 h post-training memory is fully expressed in response to the conditioned stimulus and thus these are regarded as non-lapse periods^18^.

RNA extracted from the dissected material was subjected to real-time RT-PCR in which the expression of the *Lym-NOS1AS* was analyzed using a calibrator-normalized relative quantification method. Notably, the results of the quantitative analysis showed no significant difference between the conditioned and unpaired groups in either the cerebral or the buccal ganglia at 6 h post-training, when memory formation is already not reliant on NO synthesis. Importantly, however, the same analysis revealed statistically significant down-regulation of the *Lym-nNOS1AS* in the cerebral ganglia from the 1 h and 4 h post-training conditioned groups (Fig. 4c), and upregulation in the cerebral ganglia from the 2 h post-training conditioned group. In contrast, no significant training related changes in the *Lym-NOS1AS* expression in the buccal ganglia in all tested groups of snails have been detected (Fig. 4d). Taken together these data show that LTM formation is associated with specific differential alterations in the expression of the *Lym-NOS1AS*, which are precisely timed and targeted to the cerebral ganglia.

## Discussion

One very intriguing development in contemporary molecular neurobiology has been the discovery that long NATs are abundantly expressed in the CNS^10,12,22^. This suggests that these RNA molecules could be engaged in various aspects of brain function. Therefore, an especially important task now is to understand which particular neural processes depend on the expression of NATs.

The major focus of the current study is a novel NOS-related long natural antisense RNA, which is expressed in *Lymnaea* brain. We named this RNA *Lym-NOS1AS*. It is important to note that the *Lym-NOS1AS* shares many features with another NOS-related NAT (*Mm-antiNos1*) that we previously identified in the mouse brain^20^. Both molluscan and mammalian RNAs are *cis*-encoded, non-coding, spliced and polyadenylated. Also, their antisense regions have similar sizes and locations. This remarkable evolutionary conservation suggests strongly that the NOS-related NATs are of functional importance. And indeed, we have shown earlier that the *Mm-antiNos1* RNA negatively regulates NO signaling in the brain and is likely to be involved in the regulation of neurogenesis^20^. Here we presented data that indicate that the molluscan ortholog can also participate in complex neural processes.

In the snail brain, two types of NOS-encoding mRNAs are expressed: *Lym-nNOS1* and *Lym-nNOS2*. Notably, while the similarity between the *Lym-NOS1* and *Lym-NOS2* mRNAs within the open reading frames is remarkably high (89%), their untranslated regions are unique^15^. Furthermore, the genes, from which these two mRNAs are transcribed, respond dissimilarly to single-trial reward conditioning. The expression of *Lym-nNOS1* is differentially regulated by training, whereas the expression of *Lym-nNOS2* remains stable at all measured post-training time points^15^. These data, together with the fact that the *Lym-NOS1AS* is complementary exclusively to the *Lym-nNOS1* mRNA raise the exciting possibility that this novel NAT, through the regulation of the *Lym-nNOS1* expression, acts as an important component of the pathway regulating LTM formation.

To validate this idea further, we utilized some principal advantages of our model system. Among them are an opportunity to investigate molecular processes at the single cell level. Here, we exploited the existence of a pair of serotonergic cerebral giant cells, the CGCs in the *Lymnaea* brain. Importantly, these easily identifiable cells gate the conditioned feeding response and are an essential part of the neural network involved in LTM formation^17^. Also, we have shown that the CGCs express *Lym-nNOS1* mRNA and that the expression of *Lym-nNOS1* in these cells is regulated by learning^15^. But do the CGCs contain the *Lym-NOS1AS*? To answer this important question, we conducted RT-PCR on isolated CGCs and demonstrated that these neurons express both the *Lym-nNOS1* mRNA and the *Lym-NOS1AS*. This result suggests that there is interaction between the sense and the antisense RNAs with a potential role in memory formation.

To further explore this possibility, we utilized the ability of *Lymnaea* to acquire LTM after a single appetitive conditioning trial, which allows to study conditioning-induced pathways in a precisely timed manner. For example, our previous relevant work established that there is an obligatory requirement for NO produced by NOS during the initial stages (up to 5 h following memory acquisition) of LTM formation^6^. The main goal of our present experiments was to establish whether *Lym-NOS1AS* is also regulated by learning. With this in mind, we launched a large-scale quantitative experiment, in which we measured the expression levels of the *Lym-NOS1AS* in the ‘learning’ ganglia at different time points after conditioning. Of note, the ‘learning’ ganglia contain neural circuits, which are essential for LTM formation^19^. Our quantitative studies culminated in two important observations. First, *Lym-NOS1AS* is differentially regulated by training within the learning ganglia. Namely, it is downregulated or upregulated in the cerebral ganglia at specific time points but shows no change in its expression in the buccal ganglia. Second, *Lym-NOS1AS* expression in the cerebral ganglia is transiently suppressed at 1 h and 4 h and transiently stimulated at 2 h after conditioning. Thus, the observed learning-induced changes in the *Lym-NOS1AS* ‘gene’ activity are targeted to the cerebral ganglia, where most of the NO-dependent information processing takes place during memory consolidation^15^. Furthermore, these changes are precisely timed and occur at a period when memory consolidation goes through a critical phase^18^ and NO is essential for LTM^6^.

It was demonstrated in a previous study that memory consolidation in the snail, just like in higher organisms including humans, develops through consecutive periods when the strength of the memory fluctuates resulting in intervals disrupting the continuous strengthening of the memory trace. During these intervals, the memory temporarily becomes weak and vulnerable to interference^18^. One such ‘lapse’ period was identified in *Lymnaea,* at 2 h post-training. In contrast, the 1 h and 4 h time points were identified as ‘non-lapse’ periods. Interestingly, the findings reported in the current paper reveal a correlation between the lapse/non-lapse periods and the level of *Lym-NOS1AS* expression. Specifically, the 1 h and 4 h non-lapse periods coincide with the downregulation of the NAT, whereas the 2 h lapse period coincides with the upregulation of the NAT. Thus, we can suggest that the observed suppression of the *Lym-NOS1AS*-dependent “NO break” at 1 h and 4 h post-training promotes NO production and provides a plausible explanation for the robustness of NO-dependent memory trace at these time periods. And the other way around, the detected activation of the break at 2 h post-training suppresses NO production and therefore can account for the observed temporary interruption of the NO-dependent phase of memory consolidation. Furthermore, the lack of difference in *Lym-NOS1AS* levels between conditioned and unpaired control animals at 6 h post-training also fits in perfectly with the previous conclusion that by this time the NO-dependent phase of memory consolidation is over^6^. Moreover, the cerebral ganglia are known to be involved in forming LTM. Therefore, our findings that the training-induced changes in the expression of *Lym-NOS1AS* are targeted to the cerebral ganglia further support our idea that this non-coding NAT is a component of the molecular pathway activated by one-trial conditioning.

Finally, both our everyday experience and the numerous behavioral studies show that not all learning culminates in the formation of long-lasting memories. This is due to the existence of inhibitory constraints that apply a continual brake on the molecular mechanisms the activation of which is required for LTM, such as the NO pathway. It is possible, though we currently have no direct evidence on this point, that *Lym-NOS1AS* represents an important example of such memory suppressors. Apparently, single-trial induced LTM requires this memory constraint to be absent or reduced at 1 h and 4 h following the learning event; time points representing non-lapse periods during memory consolidation. However, whether the revealed targeted and precisely timed removal of the brake provided by the *Lym-NOS1AS* is essential for or simply facilitates LTM formation has yet to be established.

## Material and methods

### Experimental animals

Specimens of *Lymnaea stagnalis* were raised in the breeding facility of the University of Sussex, where they were kept in 20–22 °C copper free water under 12 h light/dark cycle. They were fed on lettuce 3 times and a vegetable-based fish food twice a week as described previously^6^.

### cDNA library screening

A *Lymnaea* CNS cDNA library was screened using a radioactively labeled fragment of *Lym-nNOS1* cDNA^21^. A positive clone containing a cDNA insert of 2.5 kb was selected for further examination. Sequence analysis of the insert has shown that it contains a region complementary to the *Lym-nNOS1* mRNA.

### In situ hybridization

In situ hybridization of frozen sections of *Lymnaea* CNS was performed as previously described^23^. The labelled probe (5′-CACAGGA(AC)GGTATGGTGTTCT-3′) was prepared using the DIG Oligonucleotide Tailing Kit (Roche) according to the manufacturer’s protocol.

### Reverse transcription-PCR on the cerebral giant cells

The cell bodies of CGCs were identified and individually dissected from the CNS of *Lymnaea* as described previously^15^. Total RNA extracted from the CGCs (n = 10) by means of the Absolutely RNA Nanoprep kit (Agilent Technologies) was subjected to reverse transcription. The reverse transcription reaction was carried out in a final volume of 5 μl using the iScript cDNA synthesis kit according to the manufacturer’s protocol (Bio-Rad). The resulted cDNAs were amplified by means of the HotStar Taq DNA polymerase according to the manufacturer’s protocol (QIAGEN). The following primers were used: 5′-AGTTTGAGGGATGAGAACCT-3′ and 5′-AAGGGACATTACACAGAGG-3′ for detection of *Lym-NOS1* (accession number AF012531, the amplicon size is 101 bp), and 5′-GTAATAAGCGCATTTGCATAC-3′ and 5′-CCTGGTGTGAAGCTGATC-3′ for detection of *Lym-NOS1AS* (accession number MW300420, the amplicon size is 108 bp). The resulted PCR products were resolved in 2% agarose gel. The identity of the PCR products was confirmed by cloning and sequencing.

### One-trial conditioning protocol and surgical procedures

Reward conditioning was carried out using a well-established single-trial classical conditioning protocol^6,15–18,24,25^. Snails were randomly assigned to experimental (paired) and control (unpaired) groups to be given a single conditioning and control trial, respectively. Experimental animals were exposed to a solution of amyl acetate (CS, 0.004% final concentration) and 30 s later to a sucrose solution (US, 0.67%) and stayed in the mixture of solutions for 2 min. Control animals were exposed to the CS and to the US, separated by an interval of 1 h. A randomly chosen sub-set of 20 animals from each group were retained and tested for LTM formation at 24 h after the paired and unpaired trials as previously described^15^. A third group of animals was kept under the same conditions and had the same feeding regime as experimental and unpaired control snails but was not exposed to either the CS or US. This group is referred to as naive animals. At different time points (1 h, 2 h, 4 h, and 6 h) after the treatment a randomly chosen sub-set of animals (20 snails per time point) were sacrificed and the cerebral and the buccal ganglia were removed, transferred immediately to crushed dry ice and then stored at − 80° C until use.

### Quantitative real-time RT-PCR

The frozen (− 80 °C) cerebral and the buccal ganglia dissected from experimental (paired) and control snails (unpaired and naïve) (n = 20 in each group) were used to extract RNA by means of the Absolutely RNA miRNA kit (Agilent Technologies). The isolated RNA samples were treated with DNase TURBO (Ambion) to remove any traces of DNA and then quantified using the NanoDrop microvolume spectrophotometer. RNA integrity was confirmed by gel electrophoresis. The purified RNAs were copied into cDNAs using the iScript cDNA synthesis kit according to the manufacturer’s protocol (Bio-Rad). cDNAs were amplified and analyzed on the Mx3000P real-time cycler (Stratagene) using the Biotool SYBR Green qPCR Master mix (Stratech). Cycling parameters for the PCR were as follows: denaturation, 95 °C, 15 s; annealing, 52 °C, 20 s; extension, 60 °C, 20 s. We used primers 5′- GTAATAAGCGCATTTGCATAC-3′ and 5′- CCTGGTGTGAAGCTGATC-3′ for detection of *Lym-NOS1AS* (accession number MW300420, the amplicon size is 108 bp), and primers 5′-AAGGGACATTACACAGAGG-3′ and 5′-GTGTCAGTTGGAATCCTTG-3′ for detection of β-tubulin. The amount of *Lym-NOS1AS* NAT, normalized to an endogenous reference (β-tubulin mRNA) and relative to a calibrator, was calculated as 2^−ΔΔCT^.

### Statistical analysis

In both the behavioural and molecular experiments comparisons between two independent groups (e.g., unpaired and paired) were carried out using unpaired two-tailed tests. Welch’s correction was used when the samples had unequal variances. All statistical analyses were carried out using Prism (GraphPad) software. The differences were considered statistically significant at *p* < 0.05.

## Supplementary Information

Supplementary Figures.

## Data Availability

All real-time RT-PCR data generated or analysed during this study are included in the published article. All behavioral data used for statistical analysis will be available on FigShare.
