# Supplementary material for: Time dependent differential regulation of a novel long non-coding natural antisense RNA during long-term memory formation
Source: Sci Rep. 2021 Feb 11;11:3594. doi: 10.1038/s41598-021-83190-4 (PMC7878882; doi:10.1038/s41598-021-83190-4)
Supplement: Supplementary file 1 — Supplementary Figures. [file 41598_2021_83190_MOESM1_ESM.pdf]

Time dependent differential regulation of a novel long non-coding natural antisense RNA during long-term memory formation

Sergei Korneev*, Jekaterina Garaliene, Gabriella Taylor, Ildikó Kemenes, György Kemenes

Sussex Neuroscience, School of Life Sciences, University of Sussex, Brighton BN1 9QG, UK

* To whom correspondence should be addressed. Tel: +44 (0)1273 872809; Fax: +44 (0)1273 678535; Email: s.korneev@sussex.ac.uk

Supplementary Figures

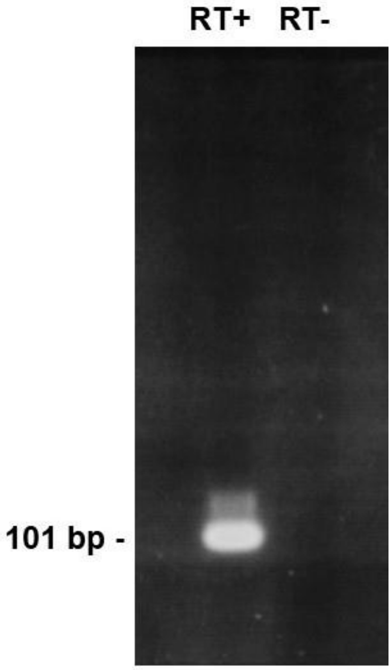

Figure S1. ***Lym-NOS1*** **mRNA is expressed in the CGC.** The results of conventional RT-PCR conducted on RNA extracted from isolated CGCs to detect *Lym-NOS1*. The 'RT+' lane shows that the PCR product of the expected size (101 bp) is detected indicating that the CGCs express *Lym-NOS1* mRNA. The 'RT-' lane represents the outcome of the control experiment in which reverse transcriptase was omitted. The absence of the amplification products in this lane is indicative that the RNA preparation was free of DNA contamination.

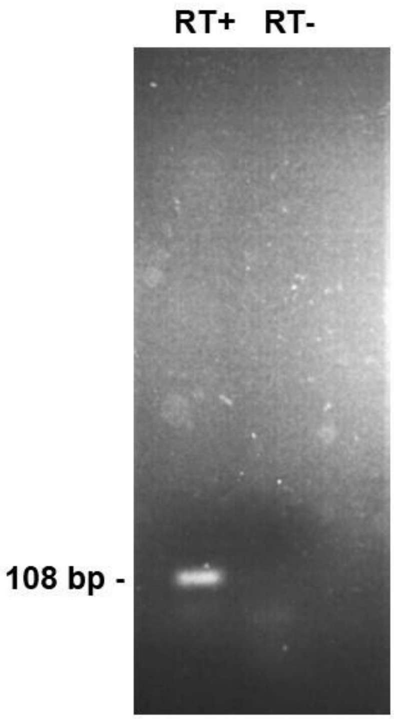

Figure S2. ***Lym-NOS1AS*** **NAT is expressed in the CGC.** The results of conventional RT-PCR conducted on RNA extracted from isolated CGCs to detect *Lym-NOS1AS*. The 'RT+' lane shows that the PCR product of the expected size (108 bp) is detected indicating that the CGCs express *Lym-NOS1AS* NAT. The 'RT-' lane represents the outcome of the control experiment in which reverse transcriptase was omitted. The absence of the amplification products in this lane is indicative that the RNA preparation was free of DNA contamination.
